# Time resolved and temperature dependence of the radiative properties of thiol-capped CdS nanoparticles films

**DOI:** 10.1007/s11051-013-2242-9

**Published:** 2014-01-16

**Authors:** N. Bel Haj Mohamed, M. Haouari, Z. Zaaboub, M. Nafoutti, F. Hassen, H. Maaref, H. Ben Ouada

**Affiliations:** 1 Laboratoire des Interfaces et des Matériaux avancés (LIMA), Faculté des Sciences de Monastir, Université Monastir, Avenue de l’environnement, 5019 Monastir, Tunisia; 2 Laboratoire de Micro-Optoélectronique et Nanostructures (LMON), Faculté des Sciences de Monastir, Université Monastir, Avenue de l’environnement, 5019 Monastir, Tunisia

**Keywords:** Organic ligands, CdS nanoparticles, Time-correlated single photon counting, Radiative recombination, Temperature, Surface states, Nanostructure, Optics

## Abstract

In this work, we present the temperature-dependence and time-resolved photoluminescence (PL) of CdS nanoparticles capped independently with three different ligands thiophenol, thioglycerol, and l-cysteine over a broad temperature range from 10 to 300 K. The respective nanoparticles sizes in the three systems studied in this work are 1.5, 4, and 2 nm as determined from X-ray diffraction (XRD). From the analysis of AFM images, it was found that the lateral particle sizes of capped CdS nanoparticles are greater than those deduced from XRD or optical absorption measurements. The aim of this study is the investigation of the impact of the organic ligands on the radiative recombination dynamics in organically capped CdS nanoparticles. From the PL study and based on the temperature-dependence and time-resolved emission spectroscopy, we conclude that the emission of CdS QDs film originates from recombination of the delocalized carriers in the internal core states with a small contribution of the localized carriers at the interface. The PL decay reveals a biexponential behavior for the entire three samples at all temperatures. One of the two exponential components decays rapidly with a time *τ*_1_ in the range 0.5–0.8 ns, whereas the other decays much more slowly, with a time *τ*_2_ in the range 1–3 ns. The weak activation energy (32–37 meV) deduced from the temperature dependence of the PL intensity suggests the involvement of shallow traps. The analysis of the experimental results reveals a relatively narrow size distribution, an efficient surface passivation, and a satisfactory thermal stability of CdS nanocrystals.

## Introduction

The optical properties of colloidal semiconductor nanocrystals (NCs) or quantum dots (QDs) have been extensively investigated over the past decades and still attract a growing interest from both fundamental and applied viewpoints, since they reflect the remarkable size-dependent electronic structure of these materials. II–VI semiconductor NCs have been shown to possess unique optical, electrical, and optoelectronic properties for a wide range of applications. Among these, CdS is a widely used substance with many advanced technological application. It is a direct band gap material of energy band gap 2.42 eV at room temperature (Bawendi et al. 1992). It is used in photodetectors, optoelectronics, solar cell applications, and biological labels (Coe et al. 2002; Greenham et al. 1996; Klimov et al. 2000; Lin et al. 2006; Michler et al. 2000). Since nanoparticles suffer from a great amount of defect states at their surface which acts as non‐radiative pathways for the excited electrons and become detrimental to the luminescent properties of nanocrystalline phosphors, it is necessary to modify that surface by using suitable capping agents which passivates the defect states and dangling bond density (Gotesman et al. 2009; Williams et al. 2009). In fact, the simple chemical treatments can have major effects on both chemical and photophysical properties of these nanostructures. Therefore, to be used in technological applications high quality nanoparticles with narrow size dispersion, surface control, and chemical and thermal stability are needed. Thiol-stabilized II–VI semiconductor particles have proven to fulfill these requirements to a large extent. Indeed, thiol ligands, which are covalently bound to the particle core instead of being loosely adsorbed to the surface, are supposed to be more stable against exchange or loss of the ligand. In this context, stable nanoparticles of different materials (CdS, CdSe, CdTe, and HgTe) have been prepared with several different thiols having different chain lengths (Thangadurai et al. 2008; Wankhede and Haram 2003).

As the overall properties of semiconductors QDs rely on the relaxation of the carriers to the ground state, the knowledge of the dynamics of charge carriers of the nanoparticles will be instructive for various applications. Moreover, as the band-edge exciton is expected to be the most important state for the relaxation of the nanocrystal, this state is very sensitive to temperature because of the location and energy distances between holes states (Bawendi et al. 1992; Califano et al. 2005; Crooker et al. 2003; Lee et al. 2005; Liji Sobhana et al. 2012; Valerini et al. 2005).

To better understand their physical and chemical properties for a future application, semiconductor NCs are usually submitted to extensive characterizations. Depending on what we are looking for, multitude spectroscopic methods may be used to probe the electronic structure and the energy levels involved in the allowed transitions, as well as the lifetimes of excited states and their respective energy relaxation channels. The main purpose of this work is to understand how the photophysical properties vary with changing the capping agents such as thiophenol (PHSH), thioglycerol (TG), and l-cysteine (Cyst). A very attention has been paid on the role of capping agent on the exciton recombination mechanism of CdS NCs and their effect on the optical properties and thermal stability of these nanoparticles was analyzed using time-resolved spectroscopy and temperature dependence of the PL emission in the range 10–300 K. Details of “Results and discussion” are elaborated in the following sections.

## Experimental

The chemicals used in this study were cadmium acetate dehydrate (C_4_H_6_CdO_4_·2H_2_O, ≥98 %, Sigma-Aldrich), thiourea (NH_2_CSNH_2_, ≥99 %, Sigma-Aldrich), thiophenol (C_6_H_5_SH, ≥99 %, Aldrich), Thioglycerol (C_3_H_8_O_2_S, ≥99 %, Sigma), and l-cysteine (C_3_H_7_NO_2_S, ≥97 %, Sigma-Aldrich). High-purity water was used for preparation of all aqueous solutions.

We have synthesized CdS nanoparticles by wet chemical route using thiourea (NH_2_CSNH_2_) and cadmium acetate dehydrate (Cd (CH_3_COO)_2_·2H_2_O) as starting material, and the three different thiol stabilizers indicated above. The typical synthesis procedure is described as follows. Firstly, 5.7 mmol of cadmium acetate dehydrate and 13.8 mmol of capping agents were dissolved in 200 mL of deionized water to obtain a solution for which the PH was adjusted from 10 to 11 by drop wise addition of 1 M solution of KOH. The solution was placed in a three-necked flask fitted with a septum and valves and was deaerated with N_2_ bubbling for 30 min. A second aqueous solution of thiourea (2.88 mmol in 50 mL) was also prepared and added drop wisely to the first one under vigorous stirring. The precursors were converted to CdS nanoparticles by refluxing the reaction mixture at 100 °C for 2 h under N_2_. This conversion is accompanied by a change of the solution color to yellow. The heating of the solution leads to the increasing of absorption at the expense of the short wavelength bands and new absorption maxima appeared at specific wavelengths suggesting the formation of some thermodynamically favorable cluster structures. Moreover, we have observed that a prolonged refluxing caused a continuous red shift of the absorption edge up to 410 nm due to continuous growth of the particles and this shift was not accompanied by a formation of new pronounced absorption maxima. To isolate the samples of CdS nanoparticles, the final solutions were concentrated down to 25 mL using evaporator. After this stage, the particles were extracted by precipitation in methanol, the solutions were stirred for 1 h, and the precipitates were filtered and dried in desiccators under vacuum.

UV–Visible absorption spectra were obtained with a DR 5,000 HACH LANGE UV–Vis spectrophotometer. X-ray diffraction (XRD) powder spectra were taken by XPERT PRO MPD Panalytical X-ray generator using Cu-Kα radiation at a wavelength of 1.542 Å. The scanning range was between 20° and 70° (2*θ*), with a step size of 0.02°. As a result, the signal to noise ratio of this measurement is very good. The PL was excited using the 375 nm line of an Argon-ion laser. The emission was dispersed by a high resolution spectrometer and detected by a high-sensitivity GaAs photodetector. Time-resolved photoluminescence (TRPL) measurements were performed using a pulsed diode laser with duration pulses 70 ps and repetition rate of 80 MHz operating at 375 nm. TRPL detections were performed by using a time-correlated single photon counting (TCSPC) board to analyze the light dispersed by monochromator and detected with a Si avalanche photodiode (APD) detector operating in Geiger mode. Low temperature studies were performed with a continuous flow liquid-He cryostat that allows temperature control in the range of 10–300 K.

The morphology on the surface and section of the films was analyzed using the atomic force microscopy (AFM). Tapping mode AFM experiments were performed in a Nanoscope IIIa Multimode AFM microscope (Digital Instruments, Veeco). Commercial etched silicon tips with typical resonance frequency of ca. 300 Hz (RTESP, Veeco) have been used as AFM probes. Roughness and section analysis of the obtained images were performed using the integrated image processing software. CdS films were produced on indium tin oxide (ITO)-coated glass substrates by drop-cast.

## Results and discussions

### Ligand dependent optical and structural study

#### XRD analysis

**Fig. 1:**
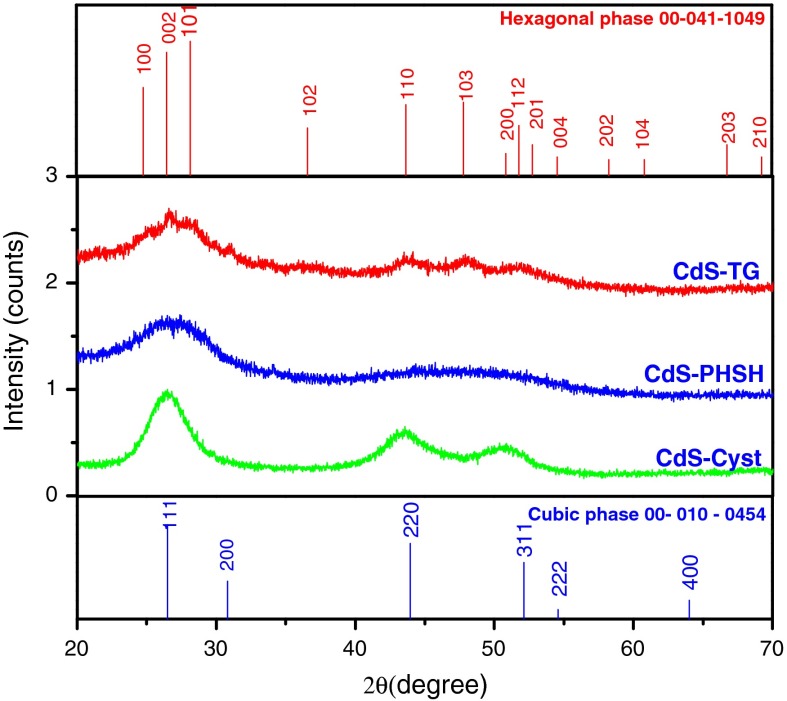
XRD patterns of CdS QDs capped with different surface ligands

The XRD diffraction patterns of CdS NCs, where the scattered intensity is plotted as a function of the (2*θ*) angle in the range between 20° and 70°, are shown in Fig. 1. The peaks in this diffractograms are found to be considerably broad indicating a very fine size, a feature typical of nanodimensional particles. The XRD patterns of CdS NCs with PHSH and Cyst powder samples are indexed with the help of JCPDS file. These diffractograms exhibit prominent broad peaks centered at (2*θ*) values of 26.46, 43.42, and 52.11. According to the JCPDS file No. 00-10-454, these peaks can be indexed to scattering from the (111), (220), and (311) CdS cubic phase planes, respectively. The peaks corresponding to (220) and (311) planes, for CdS with PHSH are further broadened due to the still smaller size of nanoparticles. This may be due to the compact packing of PHSH monolayers on the CdS nanoparticles surface which provides rigidity of self-assembled monolayers and could be responsible for preventing further growth of nanoparticles.

For CdS with TG, XRD pattern can be indexed as hexagonal wurtzite structure of CdS and they appear in good agreement with JCPDS data card No. 00-041-1049 with prominent peaks at scattering angles (2*θ*) of 24.9, 26.56, 28.27, 36.62, 43.96, 47.86, and 51.93, which could be indexed to scattering from the (100), (002), (101), (102), (110), (103), and (112) planes, respectively (Rodriguez-Fragoso et al. 2010). From the above results, we can conclude that the change of the stabilizers affects the crystallinity and the mean size of the produced nanoparticles. Indeed, the surface structure and size of the nanoparticles at any instant of time depend on the rates at which matter joins the surface or leaves it and the mobility of the surface atoms. All these processes are highly influenced by the chemical invasion of the surface layers by the sulfur of the thiols used to coat the particles and the details of any surface reconstruction by which the surface atoms of NCs decrease surface free energy (Murray et al. 1993). Indeed, the relative binding energies of ligands to different facets affect their growth rates, and consequently, control the geometry and the size of the resulting nanoparticles (Puzder et al. 2004). However, symmetry considerations favor the cubic crystal structure over the hexagonal one for the formation of a spheroidal nucleus with large number of (111) facets on the surface (Banerjee et al. 2000). We, therefore, expect the CdS nanoparticles to adopt a cubic structure at the initial stage of nucleation and transform to the bulk stable hexagonal structure during growth. Earlier study has proven that the structure of CdS NCs is size dependent. The zinc-blende structure is dominant at small diameter and wurtzite structure is more important at large diameters (Zou et al. 2011). Consequently, due to their different chemical functionalities, the different ligands used in this study may be responsible for the differences in the observed XRD patterns. By using the Scherrer’s equation *D* = 0.9*λ*/*β* cos *θ*, where *λ* is the wavelength of the X-ray radiation, *β* is the full width at half maximum (FWHM) of the intensity peak, and *θ* is the angle of diffraction, the average size of the CdS nanoparticles is determined to be 1.5, 2, and 4 nm for CdS-PHSH, CdS-Cyst, and CdS-TG, respectively.

#### Optical absorption measurements

**Fig. 2:**
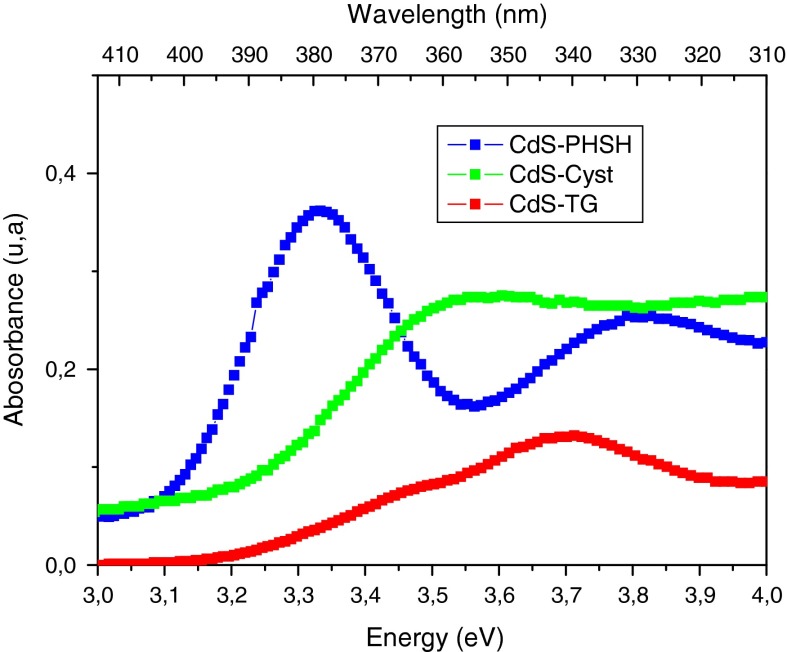
Absorption spectra of CdS NCs with different organic layers at 300 K

Figure 2 shows the optical absorption spectra of the different samples recorded at room temperature (300 K). As it can be seen, these spectra reveal different peaks where the main one which is attributed to the first excitonic transition (Tamborra et al. 2004), is located at about 360, 365, and 372 nm for TG, Cyst, and PHSH, respectively, and their corresponding absorption edges are near 395, 400 for and 406 nm. These edges clearly show a blue shift in comparison with bulk absorption edge of CdS at 512 nm (2.42 eV). This shift results from the increase in the energy gap between the valence and conduction bands of the semiconductor as a consequence of the quantum confinement of the charge carriers with decreasing particle size (de Mello Donegá et al. 2006). Based on the effective mass approximation, the average particle size of the nanoparticles can be estimated from the following equation (Brus 1986):

1 $$E_{g}^{p} = E_{g}^{b} + \frac{{\hbar^{2} \pi^{2} }}{{2R^{2} }}\left( {\frac{1}{{m_{e}^{*} }} + \frac{1}{{m_{h}^{*} }}} \right) - 1.86\frac{{{\text{e}}^{2} }}{\varepsilon R},$$

where $E_{g}^{p}$ *et* $E_{g}^{b}$ are the band gap energies of the nanoparticles and bulk CdS crystal, *R* is the average radius of nanoparticles, $m_{e}^{*}$ and $m_{h}^{*}$ are the effective masses of electron and hole, respectively, and ε is the dielectric constant of CdS. The second term is derived from the spherical confinement, whereas the third term is given by Coulomb interaction between an electron and hole. The optical gaps were estimated from the edges of the absorption spectra using Tauc relation, and the nanoparticles radii were estimated from the last equation and are found to be equal to 1.8, 1.9, and 2 nm for TG, Cyst, and PHSH, respectively.

It is clear that the crystallite sizes of CdS nanoparticles estimated from the XRD method are different from those deduced from the optical study. The reason is that the XRD measurements in polydisperse systems are weighted toward the larger particle. The result of this is a larger value of the estimated grain size. Furthermore, the inconsistency may be due perhaps to the fact that optical absorption was collected on the dissolved nanoparticles, whereas the X-ray spectra on the dried powders. In solution, particles are well dispersed, whereas in powder particles may aggregate and rearrange themselves due to electrostatic interactions. Additionally, for smaller particles, the approximations made in reaching the EMA model are no longer valid due to deviation in the estimated values (Mansur and Mansur 2011).

#### AFM images

**Fig. 3:**
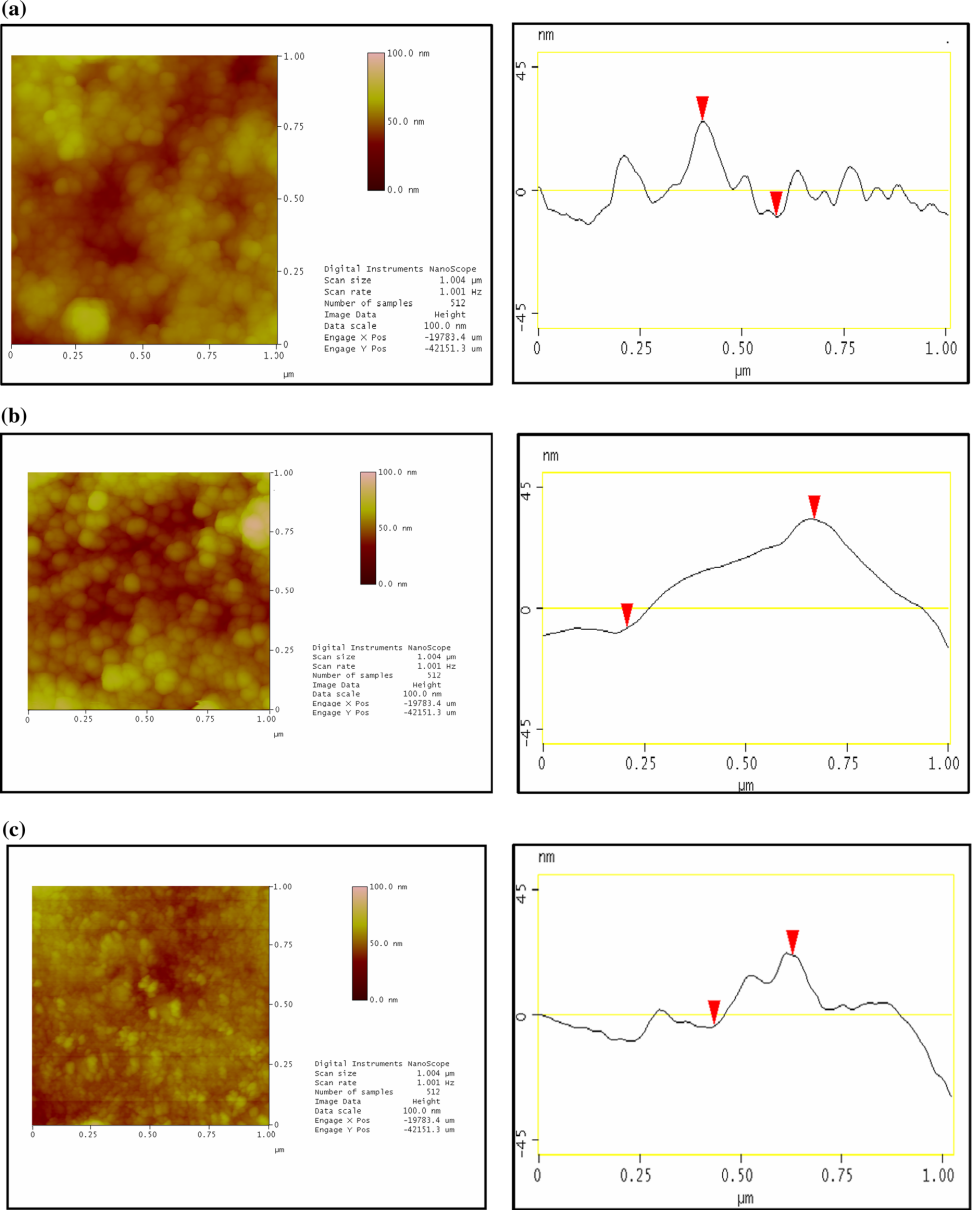
Tapping mode AFM images and section analysis of the surface of CdS thin films with different ligands. **a** CdS Cyst, **b** CdS-PHSH, **c** CdS:TG

The AFM images were recorded in the tapping mode and they are presented in Fig. 3. From this figure, the mean surface roughness of the films was found to be 20 nm for CdS-PHSH, 15 nm for CdS-Cyst, and 8 nm for CdS-TG. Obviously, the roughness increases with the particle size. The mean lateral sizes deduced from AFM experiment are in the range between 15 and 40 nm and they seem to be larger than the values calculated from the optical absorption and XRD study. This should be attributed to the intrinsic enlarging effect of the microscopic pinpoint to the measured nanoparticles, leading to overestimated dimensions (Shankara Narayanan and Kumar 2006; Nayan et al. 2013). It is also expected that XRD measurements offer the size of the crystalline domain which may be encapsulated by a layer of the amorphous material. Consequently, the size estimated from X-ray measurements is always smaller than the size evaluated from AFM measurements. Moreover, the larger particles observed in AFM image can be ascribed to the contribution of the organic capping and/or the aggregation of the smaller ones. In fact, the capping agents may not cover completely the surface of nanoparticles, so a trend for further aggregation may occur due to electrostatic interactions between particles with different sizes (Maganov and Whangbo 1994). Since the enlarging effect in the AFM images is more effective on the lateral dimension, it is expected that height information is more accurate. Therefore, we suggest that the large height deduced from section analysis of the AFM images (~50 nm) is the result of the superposition of many nanoparticles monolayers in the deposited film (Bruno et al. 2000; Nayan et al. 2013).

#### Room temperature photoluminescence study

**Fig. 4:**
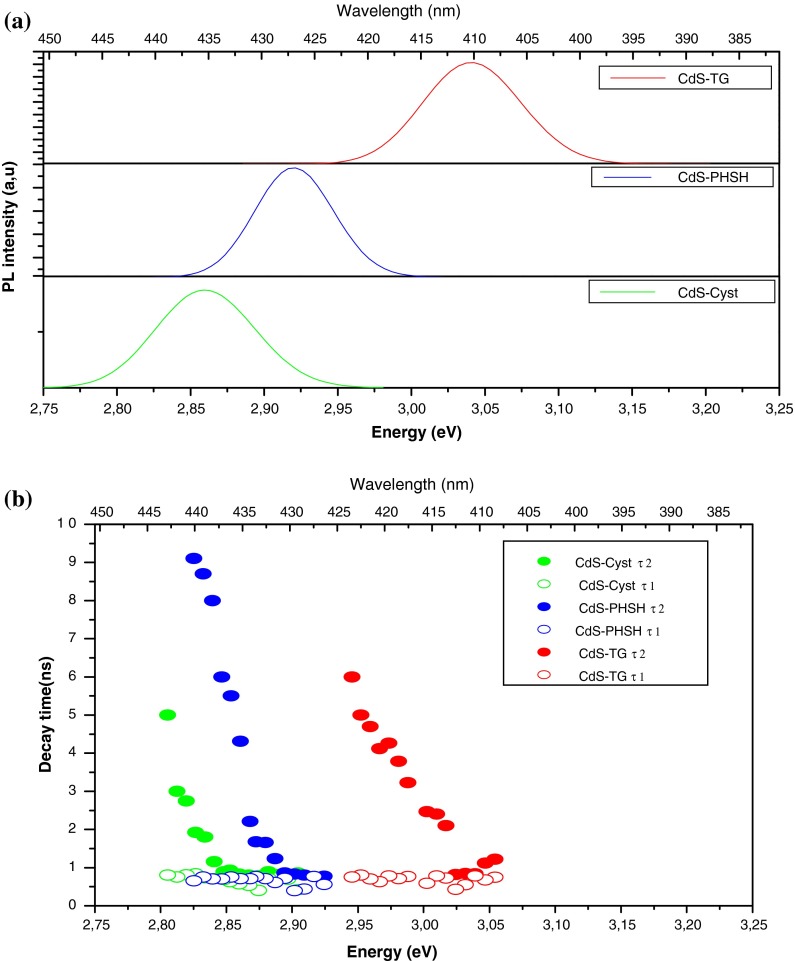
**a** Photoluminescence spectra of CdS NCs with different organic layers at 300 K. **b** The emission-energy dependence of the two decay time components

It is known that the photoluminescence (PL) studies provide information on the different energy states available between valence and conduction bands responsible for radiative recombination in semiconductors NCs. In this context, we have monitored the PL spectra for the different films of CdS nanoparticles under 375 nm excitation wavelengths (Fig. 4a). Each of these spectra reveals a narrow peak with a maximum ranging from 410 to 435 nm. The full width at half maximum (FWHM) of this emission is in the range between 25 and 60 meV for all samples indicating fairly uniform size distribution and improved crystallinity (Yang et al. 2010). As the PL energies are less than the band gap energy and by analyzing the shift from the absorption onset with the particle size, it is concluded that the main PL peak is attributed to exciton emission or/and recombinations from states involving shallow traps. Since these states are very close to the band edge, their emission is indistinguishable in the total emission spectra. Generally, the luminescence characteristic of CdS NCs usually seen in the literature consists of two emissions (Tamborra et al. 2004). The first one is a relatively sharp band having spectral width of few nm, which is highly related to the particle size and assigned to direct exciton recombination or the involvement of shallow traps. The second emission, which appears generally in the visible part of the spectrum in the 500–700 nm regions, is attributed to the recombination of trapped electrons/holes in some surface states or bulk defects. Consequently, the lack of a clear emission associated to localized surface states in the band gap reflects a good passivation of CdS particles with the thiol molecules used in this study. In fact, ligand passivation do not eliminate surface states, but shifts their energies away from the band gap center toward the electron and hole quantum-confined core energies (Li et al. 2009). Usually, in CdS, defects consist of cadmium vacancies, sulfur vacancies, interstitial sulfur, and cadmium atoms adsorbed on the surface (Schroeder et al. 1991). On the other hand, it was suggested also that thiols may generates hole traps at an energy level above the valence band of CdS NCs and it was assumed that this trap leads to a quenching of the excitation emission (Abken et al. 2009; Chowdhury et al. 2007; Liu et al. 2008).

One of the most common features of QDs is the Stokes shift Δ*S* between the positions of the PL peak with respect to the absorption one. This shift provides information about the different emission mechanisms with different dynamics which may exist concurrently. According to the literature, this shift is mostly explained based on a phonon coupling (Nirmal et al. 1994), polaron shift that results from the exciton-acoustic phonon interaction (Itoh et al. 1995), surface traps (Masumoto 1996), dark exciton, and particle size distribution (Efros et al. 1996). Additionally, it is suggested that for an inhomogeneous sample, the Stokes shift will be dominated by energy relaxation that shifts the PL peak from the first exciton absorption peak to much lower energy than its predicted zero-phonon line (de Mello Donegá et al. 2006). However, the sharp emission observed in this study reveals a narrow size distribution. Thus, we expect that the nonresonant Stokes shift can be ruled out. Moreover, the small Stokes shift measured in this study did not significantly depend on the ligands and the average particle size (0.15 eV for CdS-TG, 0.24 eV for CdS-Cyst, and 0.17 eV for CdS-PHSH). In addition, since emission from surface trap states would result in a larger Stokes shift, we expect that the luminescence observed in this work arises mainly from band-edge emission.

**Fig. 5:**
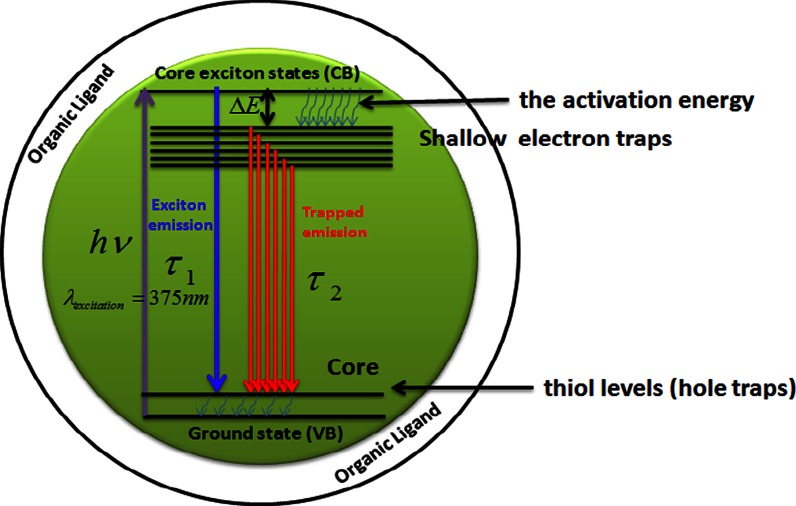
Schematic figure of the proposed relaxation mechanism in CdS NCs with different organic layers

Since the observed Stokes shift do not vary significantly with the particle size, we believe that the electron–phonon coupling has a minor contribution to this shift (Scamarcio et al. 1996). Another possible contribution may reside in the fact that absorption spectra were recorded for dilute solution, whereas the PL spectra were monitored for films of the same particles, and the red shift of the emission may be indicative to some extent of particles aggregation. Following the literature and based on the previous arguments, we assign the main contribution to the Stokes shift to the presence of shallow surface states which are mixed with the band-edge emission. The other possible source of the Stokes shift may be the electron–hole spin exchange interaction. This interaction splits the optically active exciton ground state into dark and bright states, the lowest of which is optically active (Zhonghua et al. 2003). Based on the results and discussion above, we summarized the emission bands in the scheme of Fig. 5.

#### Time-resolved photoluminescence spectroscopy

**Fig. 6:**
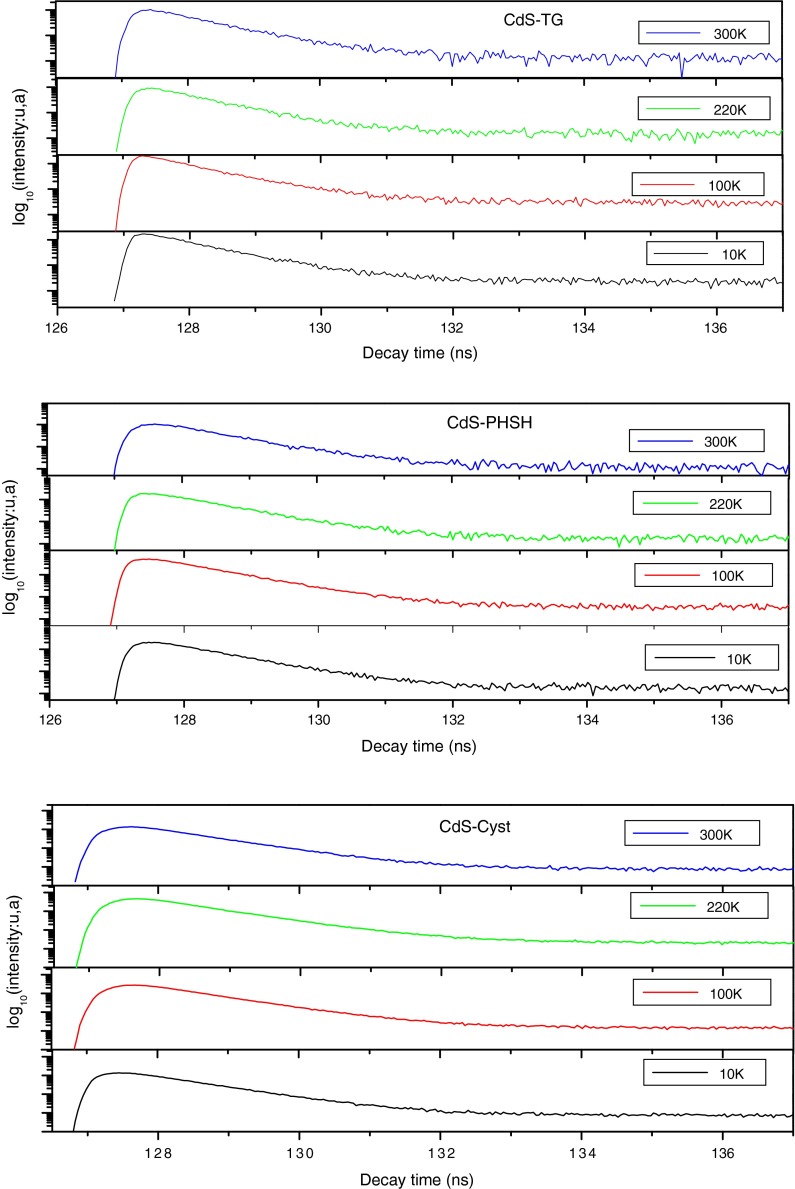
The emission decay versus temperature

To further clarify the origin of the PL bands near the absorption edge and to investigate the luminescence mechanism of CdS QDs and to determine the cause of the shift of emission peak, TCSPC experiments were performed in the picoseconds time scale. In Fig. 6, we show (RT) PL of the CdS NCs film. The film was excited at 375 nm, and the PL decay was monitored at the peak of the emission.

As a result of the complexity in the nature and the energy dispersion of the trapping sites involved and interaction with QD environment, the emission decay is usually not exponential (Xu et al. 2011). The PL decay recorded in this study for all the three samples can be well fitted by a biexponential function:

2 $$I(t) = A_{1} {\text{e}}^{{ - \frac{t}{{\tau_{1} }}}} + A_{2} {\text{e}}^{{ - \frac{t}{{\tau_{2} }}}} ,$$

where *A*_1_ and *A*_2_ represent the corresponding amplitudes of the two components at *t* = 0. One of the two exponential components decays rapidly with a time *τ*_1_ and the other decays much more slowly, with a time *τ*_2_ indicating that they come from excited states of different natures. The obtained values are in the range of 0.5–0.8 ns for *τ*_1_ and 1–3 ns for *τ*_2_. Different possible explanations for such decay behavior have been proposed (Lee et al. 2005; Passow et al. 2002). The fast PL decay component *τ*_1_ may originate from the recombination of the delocalized carriers in the internal states representing thus the lifetime of the band-edge emission decay, whereas the slow PL decay *τ*_2_ can be attributed to the recombination of the localized carriers at the surface, where both radiative and non‐radiative traps are located. However, the slight difference between the two lifetimes indicates that the emission has a nearly single-exponential decay, which suggests a high crystallinity and weak contribution of defects in the electron/hole recombination process (Na et al. 2006; Sarma et al. 2010). The narrow bandwidth in the emission spectrum also indicates essentially band-edge emission following fast intra-band relaxation due to efficient electron–phonon/hole coupling (Sarkar et al. 2007). This is consistent with the absence of clear defect peak in the emission band.

The difference in the emission decay profiles for the different thiol-capped CdS nanoparticles may occur due to the different particles sizes and the structures of the thiols used and their attachment with the CdS nanoparticles. In fact, the change of the PL decay times is the result of a competition between radiative and nonradiative processes. Hence the decrease of the fast decay channel, suggests that the fast radiative channel is plagued by nonradiative recombination. Thus, we can conclude from the obtained value of *τ*_1_ and *τ*_2_ that the good passivation is observed when PHSH is used, whereas Cyst leads to a poor encapsulation of the CdS nanoparticles. According to Liu et al. (2008), the decrease of the lifetimes in CdSe NCs involves shallow hole trapping levels introduced by the thiol ligands near the bottom of the valence band (Fig. 5).

To elucidate the origins of the two decay components *τ*_1_ and *τ*_2_, their emission-energy dependences were analyzed for CdS films with the different capping agents (Fig. 4b). As it is clear from this figure, the lifetimes of the fast PL decay *τ*_1_ remain unchanged with increasing emission energy. This suggests again that *τ*_1_ is associated with the recombination of delocalized carriers within the core states of CdS QDs (Lee et al. 2005). However, the lifetime of the slow PL decay *τ*_2_ decreases, which is a characteristic of the localization process of exciton and the rising contribution of nonradiative recombination (Strassburg et al. 2002; Passow et al. 2002). In the light of the upper discussion, we can state that after short pulse laser excitation of CdS nanoparticles, an electron exceeds from the valence band to a high state in the conduction band, and then the excited electron relaxes quickly to the bottom of the conduction band through electron–phonon scattering processes. The exciton emission arises from radiative relaxation of these electrons to the ground state and contributes to the fastest lifetime decays. However, these conduction band electrons may recombine radiatively directly or through a shallow trap states. In this case, the lifetime is extended.

### Temperature-dependent steady-state photoluminescence

#### Temperature dependence of the integrated intensity

**Fig. 7:**
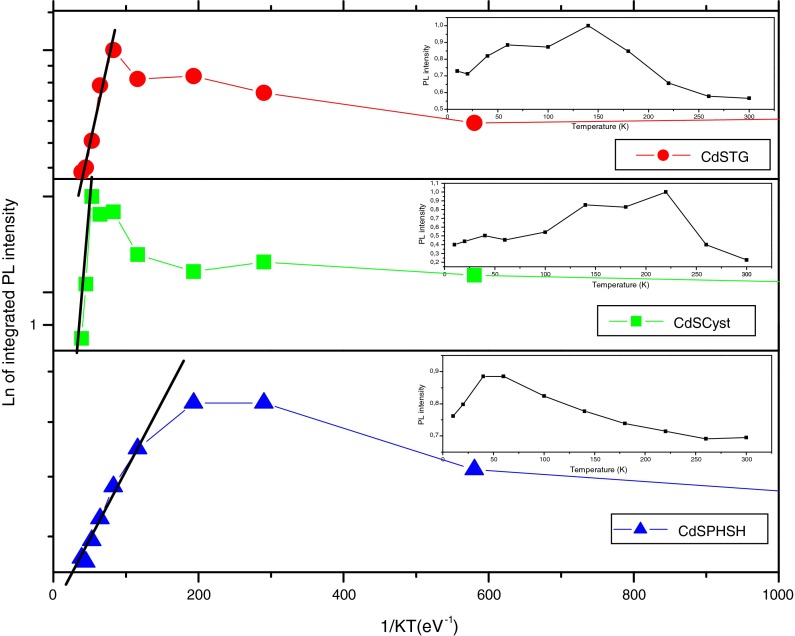
Integrated photoluminescence intensity versus 1/*kT* for CdS with different capping layers; integrated PL intensity versus temperature is shown in the *inset*

In order to investigate the organic ligands effect on the different radiative and nonradiative processes in the relaxation of our samples, we analyzed the temperature dependence of the PL intensity in the range of 10–300 K. Figure 7 demonstrates plots of the PL integrated intensity versus 1/*kT*. Depending on the ligands used in the NCs preparation, the PL spectra exhibit an increased intensity when temperature increases from 10 K to certain value before decreasing significantly owing to the thermal quenching processes. More precisely, for CdS with PHSH, the PL intensity increases slowly up to about 60 K followed by a decrease up to 300 K. This was considered to be the result of a competition between radiative and nonradiative transitions (Pendyala and Koteswara 2008). By against, for CdS-TG, the PL intensity is almost constant up to about 140 K, and it decreases up to 300 K. This behavior is due to the trapping of excited carriers from the excitonic states to the shallow levels of QDs, where temperature increases induce a gradual release of these carriers to the conduction band. Finally, the emission intensity of CdS-Cyst nanoparticles shows a drastic decrease, whereas no appreciable drop in the PL intensity has been observed for CdS with TG and PHSH, which points toward a strong suppression of the thermally activated nonradiative recombination processes. This result is probably due to the reduced density of defect states at the interface, which are commonly thought to be efficient trap centers giving rise to nonradiative decay (Morello et al. 2007; Sarma et al. 2010; Valerini et al. 2005) and is coherent with the values of the fast lifetime component found for the different ligands. The enhancement of the PL intensity when temperature increases from 10 K to a specific value to each sample indicates a thermally activated redistribution of carriers in the quantum dot in the presence of defects: with increasing temperature, carriers trapped in a defect in or around the quantum dot overcome shallow energy barriers and fall into the ground state of the quantum dot, thus increasing the PL intensity (Turyanska et al. 2007). Another way to explain this abnormal behavior is based on phase transition in the layer of capping molecules at the QD surface which removes surface states that are present in the band gap for the unrelaxed surface (Wuister et al. 2004).

Based on the theory of thermal quenching, the temperature dependence of the integrated emission intensity, *I* (*T*), can be described by the following equation (Wenzhi et al. 2012):

3 $$I(T) = \frac{{I_{0} }}{{1 + C{\text{e}}^{{ - E_{\text{a}} /KT}} }},$$

Here *E*_a_ is the activation energy (thermal quenching energy), *K* is the Boltzmann constant, *C* is a constant related to the ratio of the radiative lifetime to the nonradiative one, and *I*_0_ is the integrated emission intensity at 0 K. At higher temperature, the above equation can be written as follows (Le Ru et al. 2003):

4 $$I(T) \propto {\text{e}}^{{E_{\text{a}} /KT}} ,$$

Therefore, the value of *E*_a_ can be easily deduced from the slope of the plot of Log (*I*) versus 1/*kT* at the higher temperature region (Keith et al. 2010; Yamamoto et al. 2007).

The weak values of *E*_a_ suggest a weak trap depth and the small difference between them indicates that the shallow traps in these structures have the same origin independently of the ligand used in the preparation of CdS QDs. The smallest value of *E*_a_ is that obtained for CdS-Cyst which means that this structure has the smallest trap depth. This result is coherent with the observed thermal quenching. In fact, the shallower the trap, the larger is the thermal quenching. This result is also supported by the shape of the emission spectra of CdS-Cyst which has the narrower line width.

**Fig. 8:**
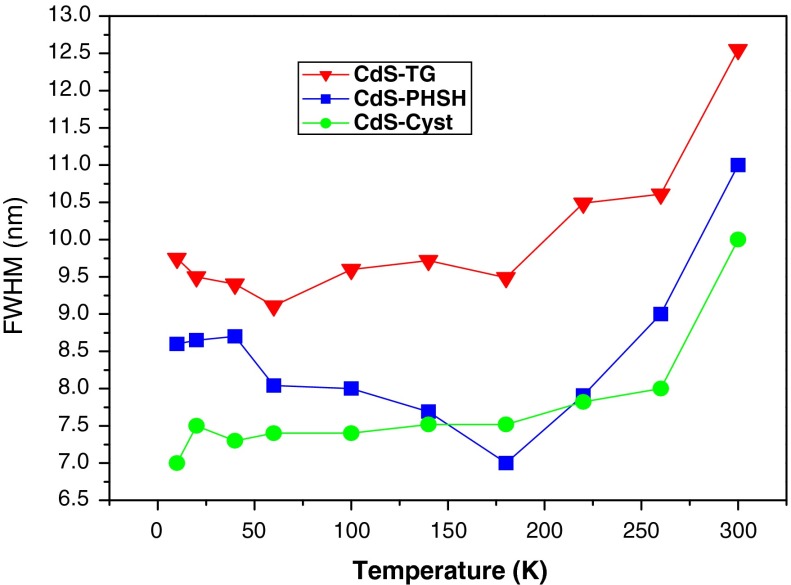
The FWHM of the PL spectra as a function of temperature

To further elucidate the effects of temperature on CdS QDs emission, the full width at half maximum (FWHM) obtained from a Gaussian fit of the PL spectra was represented as a function of temperature in Fig. 8. This figure shows a nonmonotonous behavior for all samples with a dip at 160 K for CdS-TG sample. At least, there are three contributions to the total line width; inhomogeneous broadening (due to fluctuations in size, shape, composition, etc. of the NCs), homogeneous broadening due to scattering of the exciton by optical and acoustic phonons. The inhomogeneous contribution is temperature independent and each PL curve is broad even at a low temperature as a result of multi-peak overlapping from dots of different sizes. The acoustic phonons contribution is dominant at low temperatures, while the optical phonons only contribute at high temperatures (Xu et al. 2011). In fact, at higher temperatures, phonon absorption starts to play a role in assisting the nonradiative recombination channel as shown in the decrease of PL intensity. Consequently, the usual multiple phonon coupling and thermal broadening dominate, leading to the net broadening of the peak (Xu et al. 2011).

**Fig. 9:**
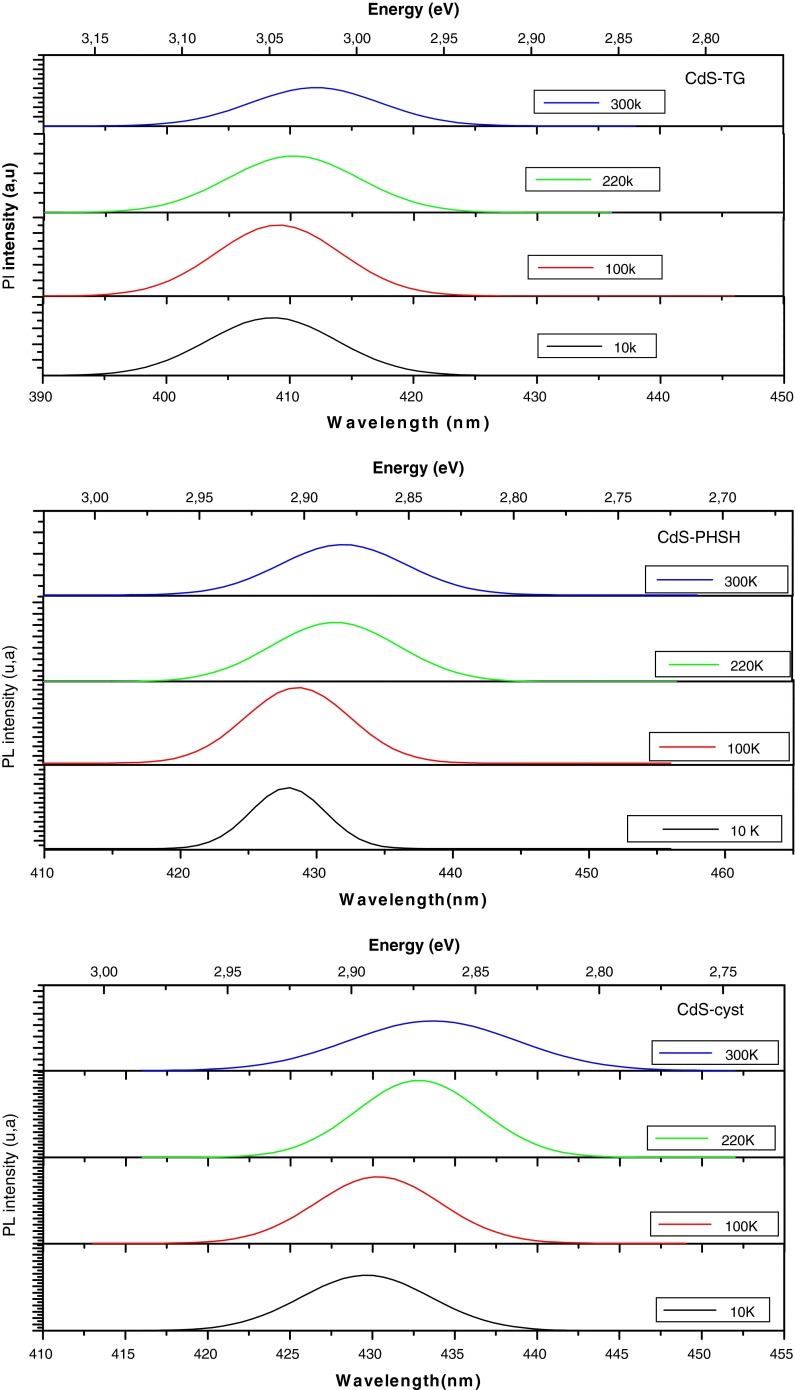
PL spectra versus temperature

**Fig. 10:**
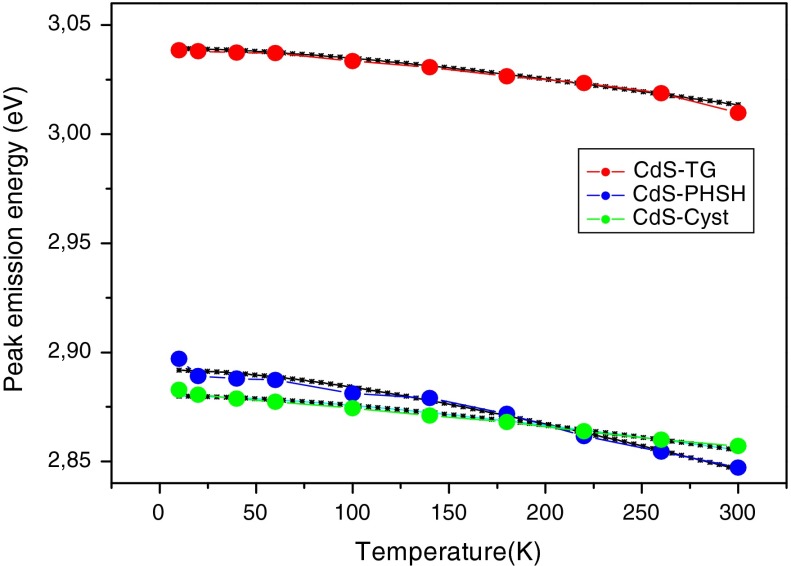
Temperature dependence of the PL emission peak energy

The PL peak blue shifts by 60–70 meV as the temperature decreases from 300 K to 10 K (Figs. 9, 10).This trend is also observed in bulk semiconductor and it reflects shrink of the energy band gap with increasing temperature due to the lattice deformation potential and exciton-phonon coupling (Jing et al. 2009). The evolution of the band gap with temperature is usually described by the empirical Varshni law (1967):

5 $$E_{g} (T) = E_{g} (0) - \frac{{\alpha T^{2} }}{(T + \beta )},$$

The *E*_*g*_(0) is the energy band gap at 0 K, *α* is a temperature coefficient, and *β* is a constant parameter related to the Debye temperature of the crystal. Consequently, we tried to fit the temperature dependence of the emission peak energy with the Varshni equation. However, the resulting fitting parameters shown in Table 1 are not similar to those of bulk CdS material (Liu et al. 2013). This suggests that the PL peak observed in this study is not purely intrinsic to the semiconductors QDs, but it involves electrons and holes near the band edge of the CdS material and it is supported by the obtained values of the activation energy of a few tens of meV which is typical of transitions between intrinsic and defect states (Wu et al. 1996). The authors of Ref. (Vossmeyer et al. 1994) observed a discrepancy with bulk CdS of the temperature dependence of the first electronic transition of 1-thioglycerol stabilized CdS clusters and they suggested that this may be due to the increase of the effective masses of charge carriers with decreasing cluster size.

**Table 1:** The physical parameters of the CdS nanoparticles obtained with different experimental techniques

Material	(*λ*_edge_)	*E*_*g*_ (eV)	Size (nm)	Structure	Size (XRD)	Emission peak (eV)	Stokes shift (eV)	Activation energy (meV)	*α* (eV K^−1^) (×10^−4^)	*β* (K)
CdS-TG	395	3.15	1.80	Hexagonal	4	3	0.15	37.5	1.6	250
CdS-PHSH	406	3.05	2.00	Cubic	1.5	2.88	0.17	35	2.8	230
CdS-Cyst	400	3.09	1.90	Cubic	2	2.85	0.24	32	1.6	244

#### Temperature dependence of the decay time

In general, the PL decay time can be divided into the lifetime for the radiative and nonradiative recombination. We take into account the temperature dependence of the nonradiative-decay rate using the Eq. 6:

6 $$\frac{1}{\tau (T)} = \frac{1}{{\tau_{\text{rad}} (T)}} + \frac{1}{{\tau_{{n{\text{rad}}}} (T)}},$$

where *τ*(*T*) is the measured average PL decay time, *τ*_rad_(*T*), and *τ*_*n*rad_(*T*) are the radiative and nonradiative recombination rates, respectively. The radiative exciton lifetime can be obtained using the following equation:

7 $$\tau_{\text{rad}} (T) = \frac{{\tau (T)I_{0} }}{I(T)},$$

where $\frac{I(T)}{{I_{0} }}$ is the experimentally measured PL intensity normalized by its value at low temperature (Byrne et al. 2006; Gurioli et al. 1991).

**Fig. 11:**
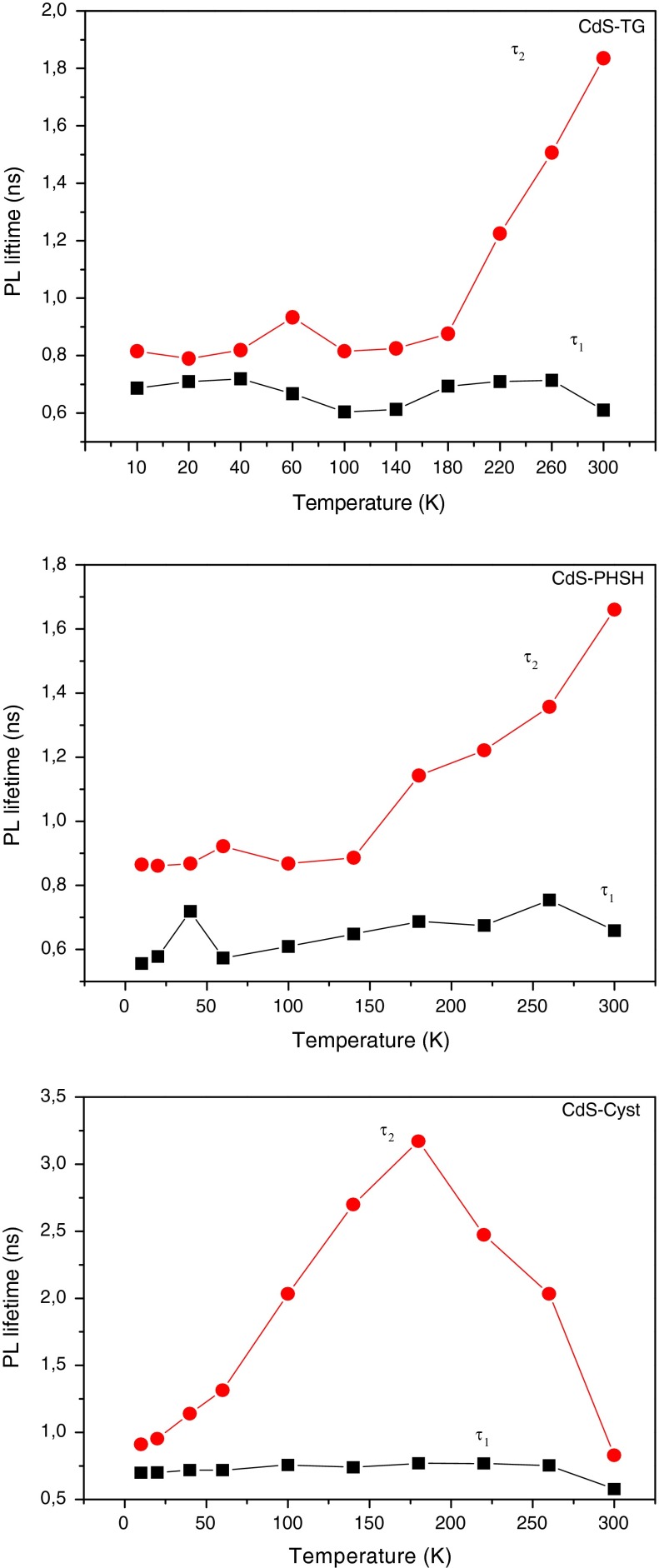
The radiative lifetimes as a function of temperature

The PL decay reveals a biexponential behavior for the entire three samples at all temperatures. The use of the above equation permitted us to calculate two radiative lifetimes corresponding to the fast and the slow components of the emission decay. Figure 11 shows that the fast component of the decay remains constant for the three samples, whereas the slow lifetime begins to increase when temperature exceeds 150 K for CdS-TG and CdS-PHSH, similar behavior was reported in reference (Raino et al. 2011). For CdS-Cyst, the slow component increases rapidly from 50 K until it reaches its maximum value at almost 180 K from which it decreases. The temperature-independent behavior of the fast component of the decay may be due to the confined acoustic phonons in the excitonic recombination and results from the superradiant emission, which is possible as long as the radiative rate dominates over dephasing (Gaponenko 1998; Lee et al. 2005). In fact, when Stokes shift is negligible, the PL kinetics is influenced by reabsorption effects, which results in a slowing down of luminescence kinetics (Gaponenko 1998). However, the absence of an important recovery between emission and absorption spectra in this study, results in weak contribution of the acoustic phonons to the exciton relaxation. According to the reference (Yang et al. 2010), the temperature dependence of the slow decay component is a result of a change of the ground state of the hole. In fact, at higher temperatures, the ground state of holes in CdS will be a mixing of *s* and *p* orbitals. The decrease in the decay time above 200 K for CdS-Cyst sample is considered to be due to an increase in the nonradiative-decay rate, which also induces a reduction in the PL intensity at higher temperatures. As it was explained above, cysteine ligand do not success to eliminate efficiently the defect states present on the surface of CdS nanoparticles which become more efficient when temperature exceeds 200 K.

## Conclusion

In conclusion, we have successfully prepared CdS QDs with different thiol ligands which present narrow emission with relatively weak Stokes shift. In order to analyze the impact of the organic ligands on the emission properties and thermal stability of CdS nanoparticles, time-resolved spectroscopy and temperature dependence study of PL were performed in the range of 10–300 K. From the analysis of the temperature dependence of the time-resolved emission, it is concluded that the observed PL is related to free excitons recombination together with emission from shallow localized states. We note, however, that the intensity of PL even at room temperature was almost that observed at low temperatures, which means that the thermal quenching effect is very small and the prepared CdS NCs present satisfactory thermal stability in the temperature range of 10–300 K. These futures make from these particles adequate candidate for several applications.
